# Schistosomiasis Pulmonary Arterial Hypertension

**DOI:** 10.3389/fimmu.2020.608883

**Published:** 2020-12-10

**Authors:** Jean Pierre Sibomana, Aloma Campeche, Roberto J. Carvalho-Filho, Ricardo Amorim Correa, Helena Duani, Virginia Pacheco Guimaraes, Joan F. Hilton, Biruk Kassa, Rahul Kumar, Michael H. Lee, Camila M. C. Loureiro, Sula Mazimba, Claudia Mickael, Rudolf K. F. Oliveira, Jaquelina S. Ota-Arakaki, Camila Farnese Rezende, Luciana C. S. Silva, Edford Sinkala, Hanan Yusuf Ahmed, Brian B. Graham

**Affiliations:** ^1^ Division of Pulmonary and Critical Care Medicine, Department of Medicine, Tikur Anbessa Specialized Hospital, College of Health Sciences, University of Addis Ababa, Addis Ababa, Ethiopia; ^2^ Department of Medicine, Butare University Teaching Hospital, College of Medicine and Health Sciences, University of Rwanda, Kigali, Rwanda; ^3^ Division of Gastroenterology, Department of Medicine, Santa Casa Hospital, Salvador, Bahia, Brazil; ^4^ Division of Gastroenterology, Department of Medicine, Federal University of São Paulo, São Paulo, Brazil; ^5^ Internal Medicine/Pulmonary Division, Medical School, Federal University of Minas Gerais, Belo Horizonte, Minas Gerais, Brazil; ^6^ Internal Medicine/Infectious Diseases Division, Medical School, Federal University of Minas Gerais, Belo Horizonte, Minas Gerais, Brazil; ^7^ Pulmonary Department, Hospital Júlia Kubistchek, Fundação Hospitalar of Minas Gerais, Belo Horizonte, Minas Gerais, Brazil; ^8^ Department of Epidemiology and Biostatistics, University of California San Francisco, San Francisco, CA, United States; ^9^ Department of Medicine, University of California San Francisco, San Francisco, CA, United States; ^10^ Department of Medicine, Zuckerberg San Francisco General Hospital, San Francisco, CA, United States; ^11^ Pulmonary Medicine, Santa Casa Hospital, Salvador, Bahia, Brazil; ^12^ Division of Cardiology, Department of Medicine, University of Virginia School of Medicine, Charlottesville, VA, United States; ^13^ Department of Medicine, University of Colorado Anschutz Medical Campus, Aurora, CO, United States; ^14^ Division of Respiratory Diseases, Department of Medicine, Federal University of São Paulo, São Paulo, Brazil; ^15^ Pulmonary Medicine, Hospital das Clinicas, Federal University of Minas Gerais, Belo Horizonte, Minas Gerais, Brazil; ^16^ Internal Medicine Department, Medical School, Federal University of Minas Gerais, Belo Horizonte, Minas Gerais, Brazil; ^17^ Hepatology Clinic, Department of Medicine, University of Zambia Teaching Hospital, Lusaka, Zambia

**Keywords:** schistosomiasis, pulmonary hypertension, neglected tropical disease, hepatosplenic, TGF-beta, type 2 inflammation

## Abstract

Pulmonary arterial hypertension (PAH) is a disease of the lung blood vessels that results in right heart failure. PAH is thought to occur in about 5% to 10% of patients with hepatosplenic schistosomiasis, particularly due to *S. mansoni*. The lung blood vessel injury may result from a combination of embolization of eggs through portocaval shunts into the lungs causing localized Type 2 inflammatory response and vessel remodeling, triggering of autonomous pathology that becomes independent of the antigen, and high cardiac output as seen in portopulmonary hypertension. The condition is likely underdiagnosed as there is little systematic screening, and risk factors for developing PAH are not known. Screening is done by echocardiography, and formal diagnosis requires invasive right heart catheterization. Patients with *Schistosoma*-associated PAH show reduced functional capacity and can be treated with pulmonary vasodilators, which improves symptoms and may improve survival. There are animal models of this disease that might help in understanding disease pathogenesis and identify novel targets to screen and treatment. Pathogenic mechanisms include Type 2 immunity and activation and signaling in the TGF-β pathway. There are still major uncertainties regarding *Schistosoma*-associated PAH development, course and treatment.

## Introduction

*Schistosoma*-associated pulmonary arterial hypertension (SchPAH) is a fatal complication of chronic schistosomiasis infection, and a leading cause of PAH-related morbidity and mortality worldwide (1, 2). The lack of understanding of pathogenic mechanisms contributes to the absence of effective therapy, resulting in worsening of the inflammatory condition and overall poor prognosis.

Schistosomiasis, also commonly known as Bilharzia disease, is a neglected tropical disease and coined to poverty (3). It is caused by parasitic flatworms (blood flukes) of the genus *Schistosoma*. The three most common species infecting humans worldwide are *S. haematobium*, *S. mansoni*, and *S. japonicum* (4), but there are also less common species including *S. mekongi*, *S. intercalatum*, *S. guineensis*, and *S. malayensis* (5). Paired male and female blood flukes reach the human blood stream, following skin invasion, where they reside for ~6 months during their lifecycle (2–4). The clinical course of the disease is divided into three stages: acute, active and chronic stage (2, 4). *S. mansoni, S. intercalatum, S. japonicum, S. guineensis* and *S. mekongi* cause hepatosplenic disease (6), a manifestation of late chronic stage and a precursor to PAH (2, 7). SchPAH has been most closely ascribed to *S. mansoni* (8), but other species have been reported (9, 10).

The schistosomiasis lifecycle is propagated by erosion of eggs through the wall of the bladder (*S. hematobium*) or intestines (*S. mansoni, japonicum*, and others) to reach the urine or feces, respectively, where the eggs are then expelled to return to the environment. Upon contact with fresh water, the eggs hatch and infect specific species of snails for each *Schistosoma* species. After a few weeks, the snails then release free-swimming cercariae into the water, which infect the mammalian host by penetrating through the skin.

Hepatosplenic schistosomiasis (HSS) is characterized by hepatosplenomegaly, liver fibrosis, portal hypertension, and esophageal varices (11), and is generally thought to be a necessary precursor state on the path to development of pulmonary hypertension (PH) (12). The chronic form of hepatosplenic schistosomiasis follows a host immune system response to eggs generated by the paired flukes and deposited into the mesenteric and portal venous system. The majority of eggs erode their way through the intestinal lumen into gastrointestinal tract to continue the life cycle. Antigenic response to eggs retained in the portal system and pre-hepatic vasculature induces inflammation and granulomas which evolve into fibrotic lesions (13). Portal shunting resulting from obstruction by eggs, granulomas, fibrosis and immune-induced vasculopathy causes portal hypertension which enables egg embolization from the portal venous system to the systemic venous system, and then into different organs with resultant consequences.

Based on current understanding, SchPAH likely results from a combination of several factors. Egg embolization into the lung *via* portosystemic shunts is associated with the development of PAH (14–17). Eggs could cause either direct mechanical obstruction or elicit an immune response which drives the vascular remodeling. Increased shear stress on the pulmonary vasculature from opening of portosystemic shunts, as occurs in portopulmonary hypertension due to cirrhotic liver disease, may also underlie SchPAH pathogenesis. Patients with schistosomiasis have a generalized, systemic Type 2 immune response, which could contribute or cause vascular disease. Using pre-clinical studies with a mouse model (see further details below), experimental disease is particularly mediated by embolic eggs causing a localized immune response and driving vascular remodeling.

The diagnostic criteria of SchPAH are challenged by the possibility of developing PH from causes other than schistosomiasis, even in those with chronic schistosomiasis. In regions where the parasite is highly endemic, personal history of infection and positive serologic studies on laboratory examination are frequent and less helpful. It is also possible to develop SchPAH without evidence of portal hypertension in patients with chronic schistosomiasis (18), although this is thought to be extremely rare and the few case reports where this occurs may actually be other PAH etiologies coincidentally occurring in those with schistosomiasis. Currently, PH is defined as a mean pulmonary arterial pressure (mPAP) > 20 mm Hg as assessed by right heart catheterization (RHC) (19), although this was recently revised from a prior threshold of 25 mm Hg used in previous definitions. Similarly, there is no consensus on a single echocardiographic threshold that defines PH, and there is often poor correlation between non-invasive echocardiography and the gold-standard invasive RHC (20). Echocardiography, however, can be used to grade the probability of PH into low, intermediate or high categories (21).

**Figure 1:**
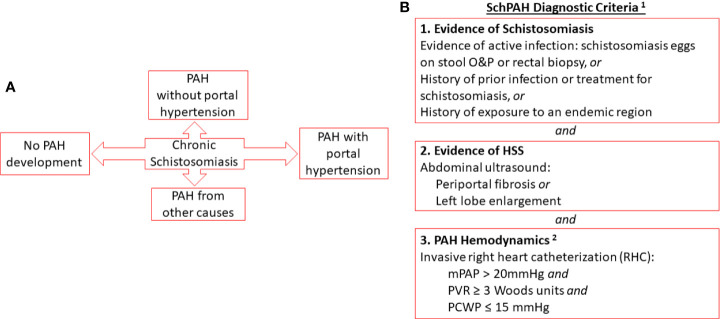
**(A)** Relationship of chronic schistosomiasis to disease complications including SchPAH **(B)**. Combined criteria for the diagnosis of SchPAH. Notes (1): It is possible for patients to develop SchPAH without HSS (2). The hemodynamic criteria are per the most recent, 6th World Symposium guidelines (Nice-2018; see Simonneau et al. ERJ 2019 [reference (19)].

Despite these limitations, the currently accepted criteria that define SchPAH (18) are a combination of: 1) PAH, defined by current criteria of precapillary PH (mPAP > 20 mm Hg and pulmonary arterial wedge pressure, or PAWP ≤ 15 mm Hg) in combination with a pulmonary vascular resistance > 3 Wood Units by RHC (19); 2) history of schistosomiasis infection, as evidenced by current or prior *Schistosoma* parasite eggs in the stool or rectal biopsy, history of prior treatment for schistosomiasis, or exposure to a region where schistosomiasis is endemic; and 3) ultrasonographic evidence of liver disease consistent with HSS, including periportal fibrosis and enlargement of the left lobe of the liver (22) (**Figure 1**).

### Epidemiology of Schistosomiasis-Associated Pulmonary Arterial Hypertension

**Figure 2:**
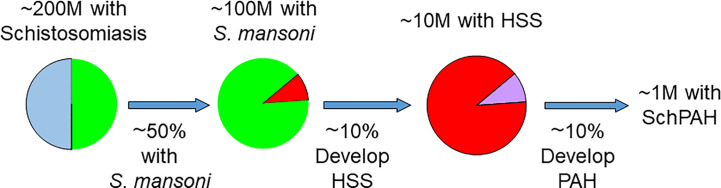
Current understanding of the epidemiology of schistosomiasis, HSS and SchPAH. With exceptions (see text), most patients who ultimately develop SchPAH have the *S. mansoni* species (accounting for about 50% of the worldwide burden of disease) and HSS resulting in portal hypertension. Please note that there is uncertainty (as discussed in the text) regarding the percentages for the prevalence of HSS among those with schistosomiasis, and the prevalence of SchPAH among those with HSS.

An outline of the epidemiology of SchPAH is summarized in **Figure 2**. The true prevalence of SchPAH is not well known. The overall prevalence of schistosomiasis infection, of all species and disease forms, is thought to be between 200 and 300 million individuals worldwide (23, 24), or about 8% of the world’s population. However, the accuracy of these data are highly limited, given that the greatest prevalence of schistosomiasis infection is in rural and economically disadvantaged areas where systematic testing and reporting is the lowest, and indeed the prevalence may be even higher.

Schistosomiasis infection encompasses infection by any *Schistosoma* species. As noted above, the three species responsible for the majority of SchPAH disease are *S. mansoni, S. hematobium* and *S. japonicum*. *S. mansoni* and *japonicum* cause hepatosplenic disease and are more clearly associated with PAH development. *S. mansoni* in particular is strongly associated with PAH. The *S. hematobium* parasite localizes to and causes pathology in the bladder plexus, and is less strongly associated with SchPAH although there are case reports (10). *S. mansoni* is the most prevalent species, potentially accounting for ~50% of schistosomiasis cases, and dual infection with *S. mansoni* and *S. hematobium* can occur (24). There are a few case reports of *S. japonicum* infection causing PAH (see below).

Individuals chronically and recurrently infected with *Schistosoma* species that cause portal disease (most notably *S. mansoni* and *S. japonicum*) may develop HSS. It has been estimated that approximately 5% to 10% of those with chronic *S. mansoni* infection develop hepatosplenic disease (24–26).

It is generally thought that HSS is a precursor stage to the development of SchPAH, although there may be rare patients who develop SchPAH without preceding HSS. Determining the precise prevalence of SchPAH in precursor populations with HSS is difficult to state with accuracy. These challenges are related to issues including which diagnostic test was used to assess disease state, as well as the evolving consensus definitions coincident with evolving understanding of disease pathophysiology.

The first Pulmonary Hypertension World Symposium in 1973 (Geneva) proposed a classification system of pulmonary hypertension based on histopathology (27). Consequently, a study by Gonçalves et al. from Belo Horizonte, Brazil published in 1995 used histopathologic criteria of schistosomiasis pulmonary arterial hypertension to assess autopsy tissue, finding histopathologic evidence consistent with SchPAH in 24 cases among 102 with HSS, for a calculated prevalence of 23.6% (28).

The second World Symposium in 1998 (Evian) proposed a classification system based on clinical features, with hemodynamic definitions: at this conference, the proposed criteria defined pulmonary hypertension as having a systolic pulmonary artery pressure greater than 40 mm Hg, corresponding to a Doppler echocardiographic regurgitant jet velocity of 3.0 to 3.5 m/s (29). Subsequent World Symposia revised the hemodynamic threshold to a mPAP ≥25 mm Hg, which was widely accepted and maintained through the third to fifth World Symposia (Venice- 2003, Dana Point- 2008, Nice- 2013). At the 6th World Symposium (Nice- 2018), the threshold was decreased to a mPAP >20 mm Hg, in the context of increasing awareness of poor outcomes in patients previously thought to have normal or borderline elevated pulmonary hemodyamics (19).

In this context, a study by de Cleva et al. from Sao Paulo, Brazil in 2003 described the pulmonary hemodynamics of 34 patients with HSS. They reported the number of subjects at a range of mPAP thresholds: 24 with mPAP > 15 mm Hg (a threshold well within the normal range), 7 (21%) had a mPAP > 20 mm Hg (the post-2018 threshold), and 4 (12%) had a mPAP > 25 mm Hg (the 2003–2018 threshold), all with a normal PAWP (30). It should be noted that schistosomiasis is not endemic in the city of São Paulo, although there are cases in the surrounding countryside; it is possible that there may be referral bias in patients that are seen at centers in São Paulo.

A study by Ferreira et al. in Recife, Brazil from 2009 used echocardiography criteria, with a threshold of 40 mm Hg. These authors found that 9 of 84 subjects with HSS had evidence of pulmonary hypertension, for a calculated prevalence of 11%. A major limitation of this study is the use of echocardiography, which provides an estimate of the pulmonary artery systolic pressure but is subject to considerable error, in the range of ±10 mm Hg or even more (31), and is additionally not capable to differentiate pre- from post-capillary PH.

A study by Lapa et al. is Sao Paulo, Brazil from 2009 evaluated 65 patients with HSS (12). Twelve of the 65 had a systolic pulmonary artery pressure >40 mm Hg on screening echocardiography (18%). Eleven of these subsequently underwent RHC (one declined); of these 11, 5 had a mPAP ≥ 25 mm Hg (the 2003–2018 threshold), but two also had a PAWP > 15 mm Hg (indicating the presence of post-capillary pulmonary hypertension). Thus only 3 of the 65 were diagnosed with SchPAH, for a prevalence of 4.6%. As noted, the mPAP threshold was subsequently lowered, so it may be that the prevalence of SchPAH would be greater with the current 20 mm Hg cutoff.

In summary, it is likely that approximately 5% to 10% of those with HSS subsequently develop PAH, depending on the specific diagnostic modality and criteria used. The current criteria [6th World Symposium, Nice- 2018 (19)] require a mPAP > 20 mm Hg which is assessed by a resting supine RHC.

### Pathogenesis and Clinical Presentation of HSS

HSS is a cause of non-cirrhotic portal hypertension in the tropics (7, 32, 33). Other causes of non-cirrhotic portal hypertension include Budd Chiari syndrome, extrahepatic portal vein obstruction, and idiopathic portal hypertension (34, 35). Most of the patients who present with features of HSS give history of exposure to schistosomiasis-contaminated water bodies remotely in childhood through occupation, domestic or recreational usage of water. It takes many years after infection for one to manifest with chronic features of HSS which include hepatosplenomegaly, splenomegaly, varices, gastropathy and anemia (36). Splenomegaly contributes to hypersplenism resulting into pancytopenia with profound thrombocytopenia. Although not common, ascites does occur in HSS and has been linked with advanced disease (36). There are known genetic susceptibility risk factors in cytokines and the TGF-β signaling pathway members that contribute to the development of HSS in those infected with schistosomiasis development (37, 38).

**Figure 3:**
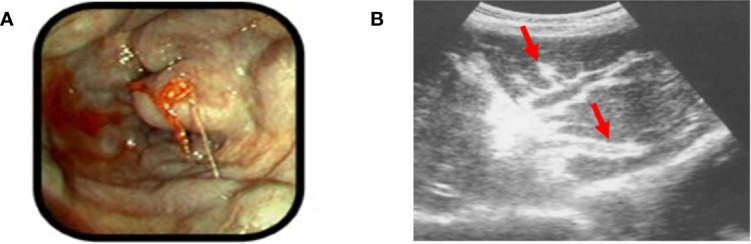
Examples of HSS **(A)**. An example of bleeding esophageal varices: note the bulging esophageal mucosa indicative of underlying varicies, with signs of active bleeding (Picture taken by ES in the endoscopy unit, University of Zambia Teaching Hospital, Lusaka, Zambia.). **(B)** Periportal fibrosis detected by ultrasound (red arrows). Reproduced from reference (39), with addition of the red arrows by the present authors to clarify the location of the pathology.

Portal hypertension in HSS is complicated by variceal bleeding (**Figure 3A**), which can be life threating and often influences observed mortality and morbidity in these patients (40–42). There are new insights suggesting that bacterial translocation may also play a role in HSS which might influence portal hypertension as is the case with cirrhosis (36, 43).

Good history and physical examination are key in making the diagnosis of HSS. Ultrasound in the clinical context has been the traditional tool to diagnose HSS and liver fibrosis specifically related to *S. mansoni* (22, 32, 44–46). The liver ultrasound classically shows periportal fibrosis (47, 48) (**Figure 3B**), and may confirm ascites if present. Enlargement of the left lobe of the liver also has a high predictive value for SchPAH diagnosis in high prevalence areas (32, 49). Magnetic resonance imaging (MRI), biomarkers of inflammation/fibrosis and portal hypertension are complementary to the abdominal ultrasound (32). In one study comparing ultrasound, histology and MRI, the presence of the characteristic periportal fibrosis, diagnosed by ultrasound, MRI or histology were associated with signs of portal hypertension and defined the severity of the disease (50). The authors concluded that imaging techniques are reliable in defining the presence of characteristic periportal fibrosis (50). Liver elastography (also known as Fibroscan) is a modified ultrasound technique for measuring liver stiffness, which may be useful in discriminating HSS from cirrhosis: the liver stiffness in higher in cirrhosis than in HSS (51, 52). Stool examination of those with HSS may show ova in active infection, but in most cases stool samples are negative as patients may have been previously infected and are now eradicated, the parasites may be old and have decreased fecundity, or the chronic fibrosis may decrease ova shedding into the stool (51). A highly specific multiplex real-time polymerase chain reaction (PCR) on stool samples in an African setting involving Kenya and Senegal was more sensitive than standard microscopy in detecting *Schistosoma* spp (53). Rectal biopsy is important especially in cases where stool examination is negative (54). Liver wedge biopsy would confirm periportal fibrosis in HSS, but it is invasive and considered to be risky due to the profound thrombocytopenia associated with HSS (50). Gastroscopy is important in making a diagnosis of esophageal and gastric varices, and gastropathy. Liver function tests are usually normal since HSS spares the liver parenchyma. Serology for schistosomiasis in endemic areas has a high rate of positivity due to widespread history of infection but does not identify the species that are or were present. Serologic testing is more helpful in the evaluation of patients from non-endemic areas. Other common causes of chronic liver disease such as viral hepatitis need to be excluded.

In general, stable patients with active HSS benefit from anti-helmintics because the treatment is generally quite safe and the diagnostic testing for active infection is imperfect (55, 56). Anti-helmintics like praziquantel help arrest disease progression, and there may be some clinical improvement, although not total resolution, of existing liver fibrosis. Beta-blockers such as propranolol are useful in primary and secondary prevention of variceal bleeding (57). Variceal banding is indicated in acute variceal bleeding, as well as in cases where prophylaxis with beta-blockers fail. Serial variceal banding is also useful in cases where beta-blockers are contraindicated. Beta-blockers are also contraindicated in patients who have subsequently developed SchPAH (58, 59). Supportive treatment such as blood transfusion in case of massive blood loss due to variceal bleeding is required. Splenectomy has shown to reduce portal hypertension and improves blood parameters in patients with HSS (60), although itself may increase the risk for PAH. Similarly, porto-venous shunts created by techniques such as a transjugular intrahepatic portosystemic shunt (TIPS) procedure may decrease portal pressures, but may also increase the risk for SchPAH development by increasing blood flow and shear stress in the lungs.

## Screening and Diagnosis of SchPAH

**Figure 4:**
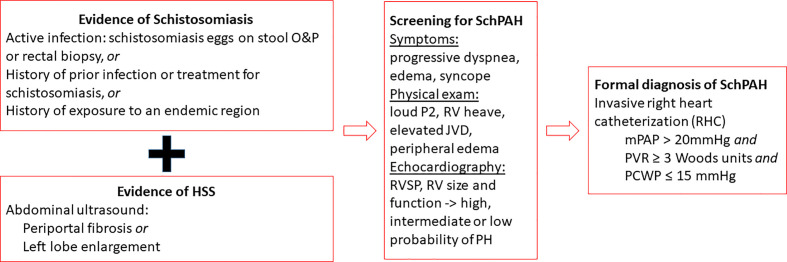
Overview of the diagnostic workup for SchPAH. The current hemodynamic criteria are from the 6th World Symposium on Pulmonary Hypertension [Nice-2018; see Simonneau et al. ERJ 2019, reference (19)].

Patients in endemic areas who present with symptoms suggestive of PAH including progressive dyspnea, fatigue, dry cough, exercise intolerance, chest pain, lower extremity edema, and syncope should be considered for SchPAH and screened for clinical disease (**Figure 4**). Other signs of PAH which can be found on physical examination include a prominent pulmonic component of the second heart sound and right ventricular heave, peripheral edema, elevated jugular venous pressure, and signs of HSS as noted above (12).

Even in non-endemic areas, epidemiologic information about risk factors for schistosomiasis exposure is important to elicit, since prior infection may give rise to SchPAH development (61, 62) despite adequate anti-helminthic treatment. Previous exposure to the parasite even in childhood, may be associated with subsequent SchPAH development in the third to fifth decade of life, depending on the time of exposure, burden of exposure, and individual predisposition presumably through other genetic and environmental risk factors (63). For that reason, the parasite eggs are often not detected in stool examination or rectal biopsy of those presenting with SchPAH.

As in HSS, abdominal ultrasonographic findings of periportal fibrosis and enlargement of the left lobe of the liver are helpful for establishing a diagnosis of SchPAH (32, 49). Detection of antibodies and/or antigens may be helpful in non-endemic areas where *Schistosoma* presence is low, and infected patients can be effectively treated with low risk of reinfection. It is recommended to use two or more assays in parallel because of limitations in serological test sensitivities (64, 65). Serologic studies are less useful in endemic areas where the prevalence of *Schistosoma* infection is high. Cardiomegaly and remarkable dilatations of pulmonary artery truncus and its branches are typical radiological findings. In particular, it has been observed that the main pulmonary artery may be more enlarged than in idiopathic PAH (IPAH) patients, suggestive of an insidious process associated with portal hypertension (66).

On this basis, individuals with signs and symptoms of progressive right heart failure, history of environmental exposure, prior treatment for schistosomiasis and/or evidence of hepatosplenic abnormalities with portal fibrosis should be screened for SchPAH. Transthoracic echocardiography is the screening modality of choice. The Doppler velocity of the tricuspid jet regurgitation can be used to estimate right ventricle systolic pressure (RVSP) using the modified Bernoulli equation. A threshold of 3.5 m/s velocity, or an RVSP of 40 mm Hg signals a high probability of PH. Other supportive findings on echocardiogram in SchPAH (and other forms of PAH) include RV and right atrium dilatation, dysfunction of the RV contraction including paradoxical motion of the septum and decreased shortening of the RV length (measured as the tricuspid annular plane systolic excursion, or TAPSE), and septal bowing.

If these signs of PH are present, RHC is performed in order to provide a direct measurement of the mPAP, assess RV function, and make sure that the left side of the heart is not compromised (which would indicate a confounding etiology, and affect management of the disease) (12). RHC is the gold standard method for the diagnosis of PH. An elevated PAP (mPAP > 20 mm Hg) along with an elevated PVR (>3 WU) in the absence of an elevated PAWP (≤15 mm Hg), confirms the diagnosis of PAH in *Schistosoma-*associated disease, as in other WHO group I PAH etiologies (12).

## Differences in Presentation and Prognosis of SchPAH Compared to Idiopathic and Other PAH Etiologies

Clinical studies in SchPAH are scarce and restricted to a few PH centers in the world, largely in Brazil (67–76). Additionally, most of the clinical data reported to date were generated in centers situated in non-endemic areas for schistosomiasis (62, 64, 65, 67–69).The classic comparator disease for SchPAH is IPAH, which occurs worldwide and has been much more widely studied despite a much lower disease burden (the incidence is approximately 1 case/million individuals/year) (77).

SchPAH occurs at an approximately 1:2 male-to-female ratio, in individuals typically between 30 and 60 years old at the time of PAH diagnosis (67–76). SchPAH patients have a similar clinical presentation compared to IPAH such as severity of disease [as measured by New York Heart Association functional class at the time of diagnosis (67, 68)] and duration of dyspnea symptoms preceding the PAH diagnosis (67). SchPAH patients also have similar symptoms as IPAH patients, such as chest pain, lower extremity edema and syncope (69). However, SchPAH patients have a more pronounced PA enlargement evaluated by chest-computed tomography compared to patients with IPAH (66).

Comparing cardiopulmonary hemodynamics at rest, as assessed by invasive RHC, patients with SchPAH generally have a less severe hemodynamic proﬁle, with lower pulmonary vascular resistance and higher cardiac output compared to patients with IPAH (69, 73). A recent systematic review of the literature including over 181 SchPAH patients with RHC data confirmed the better hemodynamic profile of SchPAH in relation to IPAH (78). However, it is important to note that most of the studies included in the aforementioned systematic review were derived from a single center, raising concerns that patient pools may overlap, potentially influencing the systematic review findings. In this context, recent data from an endemic area suggest SchPAH might have a similar hemodynamic profile at diagnosis compared to other PH etiologies (76).

Some patients with WHO Group 1 PAH have positive vasodilator response with vasodilator drug challenge such as nitric oxide – that is, have an acute drop in the mPAP ≥ 10 mm Hg and reach an absolute value ≤ 40 mm Hg with an increased or unchanged cardiac output (79). This hemodynamic profile may serve as a biomarker (80) indicating a likely clinical response to calcium channel blocker therapy and a favorable disease course. However, it is rare for SchPAH patients to have an acute vasodilator response, ranging from 0% to 3.5% (69, 71). These findings are consistent with the concept that the vascular remodeling results from fixed obstruction rather than reversible vasoconstriction.

The SchPAH mortality rate has been reported at 9.7 deaths per 1000 patients/month in endemic areas (75). The clinical course of SchPAH is believed to be more benign compared to IPAH or PAH associated with connective tissue diseases (73), even in the absence of speciﬁc PAH treatment (69). Treatment naïve SchPAH patients have overall survival rates at one, two, and three years of 95%, 95% and 86%, respectively (69). Similarly to other forms of PAH, patients with SchPAH respond to PAH-specific treatment, as demonstrated by improvements in functional class, 6-min walk distance and hemodynamic parameters (70). Additionally, treated SchPAH patients have better survival rates compared to untreated SchPAH patients (89% and 69%, respectively, at 60 months) (74). A recent systematic review of the literature confirmed the better prognosis of SchPAH in relation to IPAH (11); however, similarly to the hemodynamic findings of this systematic review, the inclusion of several studies from just one center (potential sample overlap) might have influenced the systematic review findings. Additionally, this interpretation is confounded by the lack of effective non-invasive screening methods to uniformly diagnose PAH early in its course.

In healthy subjects, oxygen consumption increases as a function of cardiac output and muscle oxygen extraction during exercise. In PAH, oxygen consumption is mainly impaired due to RV dysfunction and hemodynamic uncoupling of the RV-pulmonary arterial unit (81). These findings are suggested during cardiopulmonary exercise testing by the identification of reduced aerobic capacity, reduced end-tidal partial pressure of carbon dioxide, reduced oxygen uptake per heart beat (oxygen pulse), and ventilatory inefficiency (82). Compared to IPAH, SchPAH patients have less impaired physiological exercise responses even when matched for resting hemodynamic profiles. The better SchPAH exercise response is mainly characterized by a higher oxygen consumption and higher oxygen pulse at peak exercise, and better ventilatory response compared to IPAH (72).

Why patients with SchPAH apparently have a more benign clinical course and prognosis compared to other forms of PAH is still unclear. Furthermore, why SchPAH patients apparently have more preserved physiological exercise responses and whether this phenomenon is related to the better clinical course of SchPAH and better prognosis compared to IPAH remains unclear.

## Treatment of SchPAH

Shared pathophysiologic characteristics with other WHO Group 1 PAH etiologies opened the possibility of treating SchPAH patients with drugs targeting PAH pathways. There are 3 pathways currently targeted: the nitric oxide pathway primarily by phosphodiesterase type 5 inhibitors, including sildenafil and tadalafil, as well as soluble guanylyl cyclase stimulators including riociguat; endothelian receptor antagonists, including bosentan, ambrisentan, and macitentan; and prostacyclin analogs, including epoprostenol, treprostinil, iloprost, and the non-prostanoid IP-receptor agonist selexipag. Of these medications, due to resource limitations only a handful are available to clinicians in areas where SchPAH is endemic—particularly sildenafil as it is the least expensive. (Sildenafil is the same compound as is used in Viagra for erectile dysfunction, so it is relatively widely available for this other, more common prescribing indication.)

Preliminary data about treating these patients with PAH target therapies come primarily from open-label and/or retrospective case series and reports. One study reported the benefit of sildenafil in clinical and hemodynamic data derived from cardiac MRI in seven patients from a *S. mansoni* endemic area in Brazil. After a 3-month trial, the 6-min walk distance increased from an average of 114 to 335 m (p<0.0001), and the RV ejection fraction improved from an average of 33% to 43% (p<0.004), along with increased RV cardiac index and decreased RV mass index (83). A study from the same group of researchers reported on the efficacy and safety of 13 consecutive patients with severe SchPAH, who after 6 months of oral sildenafil had an improvement in their WHO functional class (p<0.001) and their 6-min walk distance from 121 to 394 m (p<0.0001). Using echocardiography, the average pulmonary artery systolic pressure decreased from 97 to 80 mm Hg. No significant adverse events were reported, and all patients tolerated the medication (84).

A case report described a Brazilian 35-year-old female patient with marked exercise limitation and recurrent hospitalizations due to right heart failure from SchPAH who was started on sildenafil. After six months of therapy there was an improvement in her WHO functional class and 6-min walk distance from 154 to 484 m (85).

Another study compared survival between SchPAH patients from an era before PAH medications were available and SchPAH patients receiving provider-selected medications, and found that the newer, treated series had improved survival (70). Further reviews have been published about the potential role of PAH targeted therapies in SchPAH patients (18).

The effect of anti-helminthic therapy for schistosomiasis in the lung parenchyma and vasculature has shown conflicting results in pre-clinical data. Using one experimental model (discussed further below) of schistosomiasis coupled with portal hypertension induced by portal vein ligation, there was partial improvement in the pulmonary disease following praziquantel administration (86), but using a second model with parasite infection alone there was more complete reversal of the pulmonary vascular disease following praziquantel treatment (87). Clinically, the impact of anti-schistosomiasis therapy has not been established, although some authors argue in favor of treating all SchPAH patients with praziquantel in view of the severity of the disease and low risk of harm (88). Overall, the benefit of anti-helmintic treatment likely depends on the duration of disease: in mice who have been infected for a few weeks there is likely more complete reversal than in humans who have been infected for years, in which there may be minimal clinical improvement but less rapid progression of disease without further active infection.

## SchPAH Animal Models

Development of animal models that recapitulate key aspects of the pathological processes present in human SchPAH have been critical to develop understanding of vital aspects of the host-pathogen interaction which underlie the pathophysiological and molecular mechanisms leading to SchPAH (89). Furthermore, experimental models of preclinical SchPAH are highly instrumental in identifying therapeutic and vaccine candidates. The recapitulation of the natural life cycle of *Schistosoma* in laboratory settings and the permissivity of cercarial (*Schistosoma* larval stage) infection of mice in particular has enabled the development of models of *Schistosoma*-PH (SchPH) (90, 91). (Of note, pulmonary arterial hypertension—PAH—refers to the specific human pathology involving the pulmonary arteries as occurs in SchPAH and other forms of WHO Group 1 PAH, while the more general term pulmonary hypertension—PH—refers to diseases as well as animal models more broadly affecting the pulmonary vasculature. Here the term *Schistosoma*-PH (SchPH) is used in the context of referring to animal models.)

An early model of SchPH was developed using experimental schistosomiasis coupled with portal hypertension induced by portal vein ligation in mice (86). Animals treated in this manner developed severe pulmonary schistosomiasis after 10 weeks duration. Following praziquantel treatment, mice had reversion, without fibrosis, of the peri-ovular granulomatous lesions formed in the lungs, but the arterial and arteriolar lesions were only partially improved by the treatment, with persistent segmental vascular fibrosis, narrowing, and angiomatoid changes remaining. In this study eggs were destroyed more rapidly and more completely in the lungs than in the liver.

**Figure 5:**
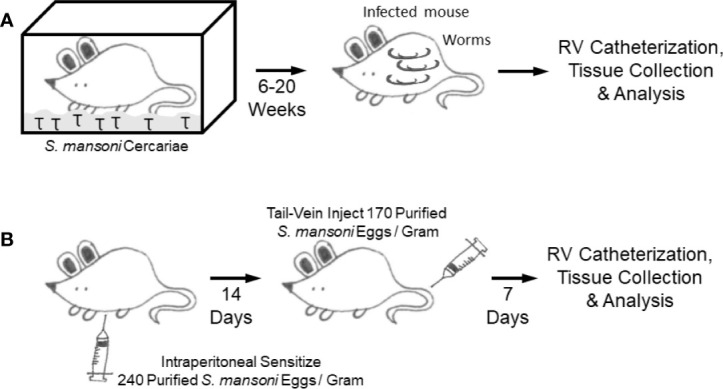
Mouse models **(A)**. Cercariae infection model **(B)**. Intraperitoneal sensitization followed by intravenous augmentation model.

Crosby et al. reported developing a murine model to study SchPH through simulating the natural mode of *Schistosoma* infection by cercariae alone (92) (**Figure 5A**), and subsequently used the same model in several publications. The mice were transcutaneously infected with *Schistosoma mansoni* cercariae in a drop of water enclosed by a metal ring placed on the shaved abdomen (93). A few weeks later egg-induced HSS developed, followed by portocaval shunt formation resulting in embolization of parasite eggs into the pulmonary pre-capillary vessels. This model recapitulated several key clinical features of human disease such as pulmonary vascular remodeling, perivascular inflammation, the presence of plexiform-like lesions (a hallmark of all forms of WHO Group 1 PAH), and elevated plasma Th1 and Th2 cytokines which positively correlated with the degree of pulmonary vascular remodeling (62, 92). However, this model did not result in higher RV systolic pressures (RVSP)—the hemodynamic parameters of PH (and one of the key criteria for SchPAH). The mice also did not consistently develop RV hypertrophy, another classical marker of experimental PH in animals (92). Other limitations of this model include inconsistency and variability in cercarial infection that might affect the development of HSS condition and resultant heterogeneity in the lung egg burden, although this can be partially overcome by using a vial to infect mice through the tail in a more controlled manner. This model also requires a long timeline for disease development (20+ weeks). There is also minor risk to personnel in handling the infectious lifeform of the parasite, mitigated through the use of gloves and other protective equipment, and ethanol/methanol can be used to rapidly kill the parasite if there is concern for exposure.

In the natural mode of infection, it is thought that portal hypertension results in shunting of eggs into the lungs, and the pulmonary pathology results from egg embolization when the eggs lodge in precapillary vessels starting a localized inflammatory response—although there are reports that SchPAH may occur without having portal hypertension or a significant parasite burden (2, 62, 69, 94). Based on the concept that *Schistosoma* egg embolization in the lungs drives Th2 inflammation and PH, another SchPH model was developed by using an existing model studying Type 2 granulomas in the lung, based on the classical immunological method of antigen sensitization followed by antigen challenge to specifically trigger organ-specific inflammation (**Figure 5B**) (95, 96). In this model, the experimental mice are first intraperitoneally (IP) sensitized with *Schistosoma* eggs, which primes the immune cells. Two weeks later, the sensitized mice are intravenously (IV) challenged with *Schistosoma* eggs administered by tail vein injection, which results in a bolus of eggs embolizing into the lungs driving localized type 2 immunity (97). RHC and histological analysis is then performed seven days after intravenous challenge. This approach recapitulates key pathologic features of human schistosomiasis PAH, including Th2 inflammation, vascular remodeling and importantly higher RVSP.

Advantages of the IP sensitization/IV challenge model include: 1) a model to study lung specific Th2 inflammation without confounding effects of other organ injury; 2) well established and high reproducibility in recapitulating key pathological features of inflammatory PAH including elevated RVSP; 3) an optimized dose of *Schistosoma* eggs, which can be modified if needed depending on the specific experimental design; 4) less mortality and shorter time (21 days) as compared to the cercariae model; 5) compatibility with inducible and global or cell specific knockout mice; 6) creation of a stereotypical inflammatory time course following egg embolization across the entire lung; 7) cost effective; and 8) non-infectious to humans or other mammals.

In the PH field, rat models are widely used. However, rats are naturally immune to *Schistosoma* infection, a phenotype which may be related to differences in the complement system between mice and rats (98). As complement may contribute to pathogenic mechanisms of PAH (99), *Schistosoma*-exposed rats have not been used to study SchPH.

Non-human primates have been used to study schistosomiasis pathogenesis (100), and other groups have studied HIV-related PH in non-human primates (101), but to our knowledge there have been no studies of PH in non-human primates using schistosomiasis. Other animal models are also possible, and there is a report that PH can occur in schistosomiasis-infected hamsters (102).

## Current Concepts of SchPAH Disease Pathogenesis

**Figure 6:**
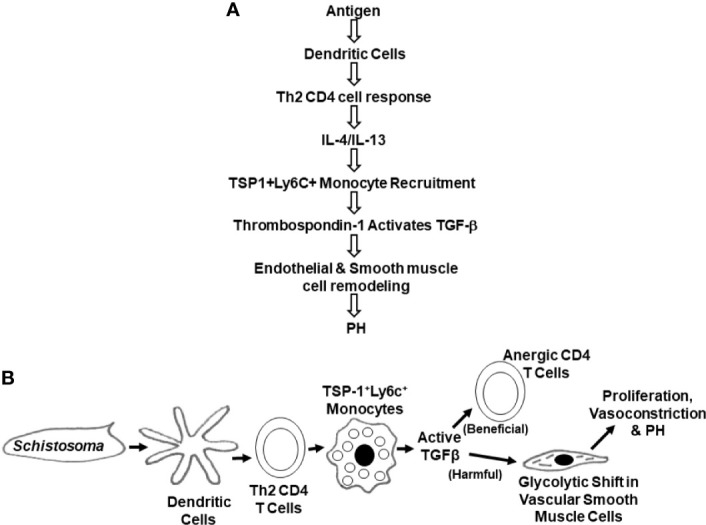
Schematics of the chain of signaling and cellular events leading to SchPAH **(A)**. Signaling events **(B)**. Cellular events.

Many studies have started to dissect the immunological events that underlie SchPH pathogenesis (1, 61, 87, 89, 92, 96, 97, 103–114). The current understanding of the signaling events is summarized in **Figure 6**.

A general concept in SchPH pathogenesis is that there is an inflammatory cascade triggered by egg antigens, which is appropriately responsive as the host immune system seeks to destroy the parasite. Within the inflammatory cascades is activation of TGF-β, a cytokine that prototypically has anti-inflammatory properties, such as suppressing CD4 T cell activation, but can induce a host of other effects. In the context of lung peri-egg granulomas, the teleologic function of TGF-β activation may be a negative feedback loop to prevent an over-exuberant immune system from causing excessive tissue injury. TGF-β has also been implicated in the pathogenesis of other PAH etiologies. Here, the pulmonary vasculature may be an unintended bystander, which is adversely affected by an off-target effect of TGF-β activation.

In clinical disease, the pathophysiology of the pulmonary vascular disease that occurs in SchPAH may involve at least three mechanisms. The first is the embolization of parasites eggs to the pulmonary arterial circulation from the portal circulation through the portosystemic shunts, following the development of HSS and portal hypertension. This embolization reaches parenchymal lung tissue as well pulmonary arteries where it causes focal arteritis with vessel destruction and plexiform lesions, such as through Th2 inflammation and TGF-β activation (14, 15). However, it has been also described that antigens of the *Schistosoma mansoni* eggs are not always present in pulmonary tissue, suggesting a second mechanism: that the inciting immunology may initially trigger vascular disease, but then separate processes take over for the propagation and maintenance of the pulmonary artery pathology in SchPAH which are now autonomous (62, 94). The third potential mechanism is the presence of liver disease (although not frank cirrhosis) giving rise to portopulmonary hypertension, another described etiology of PAH, related to high circulation flows through the pulmonary circulation (17). Of note, the pathogenesis of portopulmonary hypertension may also involve decreased clearance of toxins as is seen in cirrhotic liver disease, but not HSS. Pathologically, a characteristic vascular pathology of plexiform lesions have been described in SchPAH patients similar to those found in lung tissue from patients with IPAH and other forms of WHO group 1 PAH (115), suggestive of shared pathogenic mechanisms with other PAH etiologies. In SchPAH, a state of generalized Type 2 immunity may also contribute.

Using the mouse model, embolization of eggs alone is inadequate to develop SchPH: the mice need to be sensitized first, indicating the requirement for an adaptive immune response (96). The SchPH phenotype is also not a consequence of obstruction alone, as injection of over 10 times the number of similar-sized polystyrene beads does not cause experimental PH. Finally, mice occasionally expire on acute administration of intravenous eggs, but when this occurs, the right sided pressures are depressed rather than elevated, suggesting that there is an immune-induced circulatory collapse rather than obstruction. These data together suggest that mechanical obstruction alone is unlikely to be an adequate explanation for the elevated pulmonary vascular resistance which occurs in SchPH, and in clinical SchPAH.

The IP sensitization/IV augmentation model also establishes that *Schistosoma* egg embolization into in the lung following antigen sensitization is sufficient to drive PH, without the requirement of underlying periportal fibrosis or portal hypertension (2, 92, 108). This result suggests that clinical SchPAH could similarly occur independently of portopulmonary hypertension.

Further investigations have revealed that embolization of *Schistosoma* eggs in the lungs triggers an initial innate immune response, likely led by dendritic cells and/or macrophages. Complement activation may also be critical. Egg antigens are subsequently presented by antigen presenting cells (APCs) like dendritic cells to CD4 T cells, resulting in the production of Th2 inflammatory cytokines including IL-4 and IL-13 (97, 108). Other cells such as eosinophils and basophils are present and may contribute to production of these cytokines, but in the liver fibrosis model eosinophils and basophils are dispensable (116, 117) so they may well be dispensable in the lungs and SchPH pathogenesis as well.

Dendritic cells are critical APCs that likely orchestrate the transition from innate to adaptive responses through modulating effector T cell responses in SchPH. In SchPH, it appears after exposure to IP egg antigens that CD11b^+^ DCs migrate into mediastinal lymph nodes (based on our unpublished data), where they present antigens to naïve T cells and prime them into effector CD4+ T cells to elicit a Th2 adaptive immune response. Intravenous egg challenge then deposits antigens in the lung vasculature, where there is local homing and activation of CD4 T cells in the parenchyma, starting a cascade of Type 2 inflammation.

*Schistosoma-*exposed mouse lung tissue contains higher levels of IL-4 and IL-13, while mice with deficient Type 2 immunity by combined deletion of IL-4 and IL-13 are protected from SchPH (108). This protection was not observed in single IL-4 or IL-13 knockouts, probably due to redundant effects in their shared IL-4Rα/IL-13Rα1 receptor. A related recent study reported that paclitaxel, a chemotherapeutic agent, protects from *Schistosoma*-induced PH by dampening Th-2 inflammation (110). Reinforcing the clinical relevance of these findings, there are higher staining intensities for IL-13, IL-4Rα, phospho-signal transducer and activator of transcription factor 6 (a key target of IL-4 and IL-13), and periostin (another target of IL-4 and IL-13 signaling) in autopsy lung tissue from patients with SchPAH (108).

To explore the role of adaptive immunity in SchPH, *Rag^-/-^* mice (which lack mature B and T cells) were found to be protected from *Schistosoma*-induced Type 2 inflammation and PH. However, these mice regain the inflammatory PH phenotype after reconstitution with CD4+ T cells from wildtype donor mice (97). In contrast, we observed that blocking the Th2 function of donor CD4+ T cells by reconstituting the *Rag^-/-^* with CD4+ T cells harvested from *Il4* ^-/-^;*Il13* ^-/-^ double knockout mice protected the recipients from *Schistosoma*-induced Type 2 inflammation and PH. To elucidate the role of antigen priming, transferring CD4 T cells from mice previously sensitized to *Schistosoma* eggs was sufficient to convey the sensitization phenotype to the recipient *Rag^-/-^* mice, who then were able to develop PH after IV egg challenge only. Collectively, these data demonstrate that Th2 CD4+ T cells are necessary and sufficient to induce PH.

These Th2 cytokines then signal the bone marrow compartment to release and recruit classical Ly6c^+^ monocytes into the lungs, where they transform into interstitial macrophages (IMs). In contrast, *Il4* ^-/-^;*Il13* ^-/-^ double-knockout mice were found to have substantially fewer Ly6C^+^ monocytes, indicating that this is a Th2-dependent response (89). Corroborating these findings, it has been demonstrated that CX3CR1 plays a role in modulating monocyte recruitment, macrophage polarization, and smooth muscle cell proliferation in the lung using a hypoxia model of PH (118).

These IMs produce a key protein, thrombospondin-1 (TSP-1), which is a biological activator of TGF-β; in this disease, possibly activating the TGF-β1 isoform in particular. Following localized TGF-β activation, pathologic TGF-β signaling through the canonical Smad2/3 pathway results in inflammation and PH-related pathology (89, 109). The necessity of this pathway is demonstrated by protection from SchPH by blocking TGF-β signaling through techniques such as neutralizing antibodies, TGF-β receptor inhibitors, and Smad3 deficiency (109). TGF-β signaling can also contribute to vasoconstriction through non-canonical signaling such as RhoA/Rho-kinase, which is also observed in SchPH mice (109, 113).

A downstream effect of TGF-β that has been investigated is its role in inducing a metabolic shift in pulmonary artery smooth muscle cells (PASMCs). It has been previously observed that in PAH, the PASMCs undergo increased glycolysis, similar to the anaerobic glycolysis or “Warburg” effect observed in cancer cells (119). Using stable isotope metabolite studies, activated TGF-β was found to be sufficient to induce a glycolytic shift in PASMCs (120), which may facilitate a pathologic state of increased proliferation, hypertrophy, vasoconstriction, and apoptosis resistance (121).

TGF-β signaling is also important in the liver pathology of HSS. Type 2 inflammation and TGF-β are associated with liver fibrosis in schistosomiasis (122). Mice with heterozygosity for a TGF-β receptor which is mutated in many forms of heritable PAH, BMPR2, had evidence of worse SchPH compared to wildtype mice, a phenotype mediated by worse liver pathology and increased shunting of eggs to the lungs (114).

Of note, multiple intervention steps such as deleting IL-4 and IL-13 in the CD4 T cells or in the bone marrow (BM) compartment, or deleting TSP-1 in the BM compartment attenuates inflammation and PH phenotypes in TGF-β dependent manner (89, 97, 108, 109). These data suggest that modulating TGF-β could be a potential therapeutic target to treat *Schistosoma*-PH. The significance of the outcome of murine model is reinforced by the report of higher serum level of TGF-β in patients with SchPAH compared to those with HSS (123). Overall, the *Schistosoma* model system seems highly pertinent to understanding the signaling axis between Th2 inflammation, TGF-β signaling and pulmonary vascular disease that connects *Schistosoma* exposure to the subsequent development of SchPH. Some of these mechanisms are shared with other PH models, as blocking TGF-β signaling is also protective in hypoxia-exposed mice and monocrotaline-exposed rats (109, 113, 124, 125): TGF-β signaling may represent a final common pathway in PH pathogenesis, which is activated by different routes such as Type 2 inflammation activating TSP-1 in schistosomiasis.

## Schistosoma japonicum VERSUS Schistosoma mansoni

As was noted above, the two primary *Schistosoma* species that infect humans and cause hepatosplenic disease are *S. japonicum* and *S. mansoni*, but *S. mansoni* is the dominant cause of SchPAH. Comparison of *S. mansoni* and *S. japonicum* may thus identify key parasite and host factors that mediate the pathogenesis of SchPAH.

Geographically, *S. mansoni* and *S. japonicum* are endemic in different locations around the world: *S. mansoni* is endemic to regions along rivers and coasts in Sub-saharan African countries, South America (primarily in Brazil), and the Caribbean, while *S. japonicum* is endemic in waterways in China and South East Asia (18, 126, 127). Moreover, due to geographical distinction, both species infect different intermediate snail genus, in which *S. japonicum* infects the genus *Oncomelania*, while *S. mansoni* infects the genus *Biomphalaria* (128). Due to this geographic separation, it is unlikely that any one individual would be simultaneously infected by both species. In addition to geographical distinction, the species differ in morphology: *S. japonicum* eggs are round and *S. mansoni* eggs are oval (129). S*. japonicum* worm pairs are more fecund than *S. mansoni* worm pairs, laying about 3,000 eggs per day compared to 800 per day (130, 131).

Several studies have compared the immunity and pathology that results following chronic infection by *S. mansoni* or *S. japonicum*. In infection by both species, there is initial Type-1 immunity characterized by increased production of IL-1, IL-12, and INF- γ, triggered by worm antigens. Subsequently, the dominant inflammation phenotype shifts to Type-2 immunity caused by *Schistosoma* egg antigens, characterized by the elevation of IL-4, IL-13, and TGF-β (108, 110, 132, 133). Several studies have investigated specific proteins released by *S. mansoni* eggs that drive the Type 2 immune phenotype. In particular, there is a specific glycoprotein ribonuclease called omega-1 that has been shown to be necessary and sufficient *in vitro* for activation of Th2 CD4 T cells (134). The equivalent protein in *S. japonicum* is CP1412, although the protein sequence homology between Omega-1 and CP 1412 is low at about 40% (135).

In response to egg deposition, both *S. mansoni* and *S. japonicum* form peri-egg granulomas, although the *S. japonicum* granulomas around single egg are smaller when compared with *S. mansoni*. This could be due to higher macrophage and eosinophil composition found in *S. mansoni* granulomas compared to *S. japonicum* granulomas, which results in the production of different cytokine profiles (131). Interestingly, granulomas formed around clusters of *S. japonicum* eggs (this species’ eggs tend to cluster) are larger and dominated by neutrophils and hepatic stellate cells which suggests that the pathology due to *S. japonicum*’s inflammatory response may be more severe than *S. mansoni* (131, 136)–a paradoxical relationship compared to the incidence of SchPAH. However, S. mansoni may drive more Type 2 immunity than S. japonicum, or a slightly different combination of cytokines and cells, and differences in TGF-β activation are unclear.

Between the two, *S. mansoni* is highly associated with the development of SchPAH, but there only case reports of SchPAH occurring following *S. japonicum* infection (137). Why *S. japonicum* does not cause SchPAH as often is not known. In a prospective case series published by Watt et al (137). in 1986, 65 consecutive patients hospitalized with *S. japonicum* infection were evaluated, with 43 patients found to have evidence of HSS (32 with severe liver dysfunction and 11 had portal hypertension without liver dysfunction). Of these 43 only one patient (2%) had PAH: an incidence likely much lower than PAH in those with *S. mansoni*-induced HSS. The study suggested that the rarity of developing PAH due to *S. japonicum* could be related to the fact there is less inflammatory response around the single *S.japonicum* egg in the lungs than the liver, and the eggs dissolves quickly due to the smaller peri-egg granuloma size when compared with *S. mansoni*.

## Future Directions and Areas of Active Investigation

The abovementioned heavy disease burden of SchPAH at the global scale and the lack of targeted therapies underscore the importance of recognizing current knowledge gaps and addressing them by utilizing appropriate investigational tools. The various mechanisms by which schistosomiasis can generate and accelerate PAH introduce complexity in understanding the disease and is an area of active investigation. Areas of proposed emphasis are outlined in **Table 1** and summarized below. At least four potential inter-related mechanisms are implicated: host genetics, *Schistosoma* species infecting the host, preliminary development of HSS, and multiple candidate inflammatory pathways.

**Table 1:** Summary of areas that are priorities for research in SchPAH.

Areas of Inquiry	Examples of Specific Questions	Methods of Investigation
**Epidemiology**	What are the incidence and prevalence of SchPAH? What are risk factors for development and progression of SchPAH? What percentage of patients with HSS develop SchPAH?	Preliminary findings from retrospective data, especially for rare outcomes. Clinic-based prospective studies conducted in endemic areas can enrich cohorts for exposures and endpoints. Population-based prospective observational cohorts, stratified by risk.
**Pathogenesis**	What innate immune system components are proximal triggers for SchPH development? What are the roles of adaptive immune system components, such as B cells and regulatory T cells, in SchPAH pathogenesis? Why does human SchPAH develop and progress even after the parasite is eradicated from the host?	Mechanistic animal models using pharmacologic inhibitors and transgenic approaches will allow precise dissection of the signaling pathways that lead to SchPH. Animal models coupled to studies of human biospecimens, including tissue and blood biomarkers, will be useful for identifying pathogenic mechanisms.
**Diagnosis**	What are the sensitivity and specificity of echocardiography metrics for diagnosis of SchPAH? What are the utilities of other imaging modes in screening, diagnosing, and monitoring SchPAH? Are there other clinical markers or biomarkers such as blood tests that are useful for screening for or diagnosing SchPAH?	Diagnostic studies require gold standard and candidate diagnostic metrics to be available in a spectrum of individuals with and without SchPAH. Candidate diagnostic metrics that are weakly associated with SchPAH status may provide stronger evidence if measured serially. Noninvasive screening methods are justified in at-risk individuals and can lead to earlier diagnoses.
**Treatment**	Are there specific points in the signaling cascade that are safer to target in populations at risk for recurrent schistosomiasis infection, such as TSP-1? Do conventional PAH treatments work in SchPAH to the same degree as in IPAH? What are drug targets in SchPAH that address the underlying pathogenic mechanisms, such as HSS versus other pathways to SchPAH? Are there specific targets in SchPAH that would also benefit other forms of PAH?	Pre-clinical animal models are useful for understanding disease mechanisms and identifying potential prophylactic and therapeutic approaches. Identification of biomarkers shared or different between patients with SchPAH and other PAH diseases may reveal therapeutic targets that are beneficial in one or multiple etiologies.

In patients with HSS, fibrosis-induced portocaval shunting increases blood flow through the lungs and shear stress, similar to the pathophysiology that occurs in portopulmonary hypertension. It remains unknown why only a subset of patients with HSS progress to SchPAH. One possibility is that genetic backgrounds of human hosts determine how the pulmonary vasculature reacts to the parasitic infection. This phenomenon has been observed in patients with “idiopathic” PAH, wherein genetic variants can drive or exacerbate PAH. For example, TGF-β signaling and, in particular, bone morphogenetic protein receptor type II (BMPR2) mutations have been established as risk factors of PAH (138–140).

In addition to the innate heterogeneity of human risk and response, the nature of *Schistosoma* infections may play an important role. The incidence of SchPAH caused by non-*mansoni* species of *Schistosoma* has not been extensively studied, and it is conceivable that species differ in antigen expression – including extent of obstruction of the blood flow by embolized eggs, degrees of vascular inflammation and proliferation of pulmonary arteriopathy in the human lungs directly triggered by *Schistosoma* egg embolization, and post-infection risk of systemic Type 2 inflammation (111, 141). The number and frequency of *S. mansoni* reinfections also likely influences the resulting disease phenotype; however, practical means of documenting infection episodes and causative species are not yet clear. There may also be other infectious and non-infectious environmental factors, including comorbid conditions and patient behaviors, that affect SchPAH risks.

Another critical aspect of SchPAH pathogenesis is the observation that SchPAH persists and progresses despite eradication of the parasite from a host; that is, this antigen-triggered disease progresses later in its disease course independent of antigenic stimulation. While it has been observed that Th2-inflammation and TGF-β signaling are critical for developing the PH phenotype in the mouse model of SchPAH (89, 97, 108), whether these signaling pathways are pertinent to human SchPAH and what stimulates them in the absence of active infection are unclear. For example, TGF-β is known to be able to stimulate its own activation, potentially leading to a feed-forward loop that is independent of the initial triggering event (124).

It is likely that each of these processes contributes to the disease pathogenesis, but their relative contributions are presently not known. Approaches to understanding these issues will include dissecting the independent contributions of each of these factors. The importance of making these distinctions is supported by the concept that clinical benefit of currently available therapies, such as vasodilators and immunosuppressants, will vary depending on the predominant mechanism driving SchPAH. Based on the inflammatory nature of the parasitic infection and the role of Th2-inflammation in mouse studies, pulmonary vasodilators with anti-inflammatory properties like prostacyclin appear to be potentially attractive candidates (142). A phase 2 trial of sotatercept targeting the TGF-β pathway in other forms of PAH recently completed with promising initial data (NCT03496207), showing a significant reduction in pulmonary vascular resistance, and also an improvement in 6-min walk distance (143). Given the likely role of TGF-β in the pathogenesis of SchPAH, sotatercept may be effective in treating SchPAH patients too. Identification of relevant genetic variations in SchPAH patients may enable similar therapeutic approaches to address these targets.

True effects of anti-inflammatory therapies in human SchPAH, however, are unknown. An example of the clinical significance of the multifactorial nature of disease pathogenesis is underscored by studies of systemic sclerosis-induced PAH (SScPAH). Systemic sclerosis is known to cause PH of multiple etiologies and groups as defined by the World Symposium classification system (Group 1 PAH, and group 3 PH *via* interstitial lung disease, plus possible direct involvement of the RV), and this disease heterogeneity is believed to underlie the poor treatment response and the high mortality of SScPAH patients (144). Therefore, elucidating the clinically most relevant pathogenetic mechanisms in SchPAH remains an important task.

Discovering these unknown aspects of SchPAH pathogenesis requires the ability to generate sound hypotheses from patient-derived samples, readily test the hypotheses in animal models, and translate the findings back to clinical settings. This multifaceted research approach requires study designs to be tailored to the questions being asked, but also practical and feasible in regard to the outcome frequency and resources of the settings in which they will be implemented. In pre-clinical models, pathogenic effects can be studied using experimental designs that induce or block individual factors. In clinical studies, distinctions can be made through careful phenotyping of subjects to enrich for cohorts with HSS and/or SchPAH, and correlating PAH development with specific genotypes and phenotypes.

Initial epidemiologic studies can be conducted using retrospective clinical data, although the medical records available may be incomplete and follow up inconsistent; an estimate of the proportion of SchPAH patients under pulmonologists’ care who have a history of HSS could be obtained this way. Autopsy studies of banked tissue, to compare differential consequences of *Schistosoma* infection across species, provide another example. To uncover candidate pathogenic mechanisms, studies could use a cross-sectional design to evaluate biomarkers derived from blood or urine for their relationships with well-recognized prognostic scores that are based on clinical and hemodynamic measures.

Subsequent prospective observational studies could evaluate trends in biomarkers in relation to trends in prognostic scores, and the effects of conventional treatments on these trends. Prospective observational data being generated for clinical purposes allows enrichment of distinct at-risk pools, such as to study risk factors for incident disease and disease progression (e.g., individuals diagnosed with HSS versus individuals with SchPAH-associated symptoms, or by level of BMI). Prospective collection allows opportunities to increase data quality, by systematically timing visits among all patients across collaborating centers, and adding metrics of particular interest, such as patient-reported outcomes (PRO).

As mechanistic studies reveal pathways, clinical trials can be used to investigate therapeutic approaches. In SchPAH, early trials would likely confirm that conventional PAH treatments validated for other PAH etiologies have similar treatment effects in SchPAH. Subsequent studies would evaluate effects on target pathogenic mechanisms which may be shared with other PAH etiologies (i.e., TGF-β signaling, with an agent like sotatercept) or mechanisms that are thought to be unique to SchPAH (i.e., potentially Th2 inflammation and TSP-1 expression on Ly6c^+^ monocytes).

As the field moves forward, it is critical to consider the practicality of implementing novel diagnostic and therapeutic modalities in the resource-limited settings where SchPAH is endemic. Directly adopting practices that are being used for other types of PAH in developed countries will likely not be effective. For example, in certain parts of the world the infrastructure is not currently available to perform RHC on all patients who are suspected of having PAH, although it is the gold standard diagnostic test for PAH and required to ensure that left heart dysfunction is not present (145). Similarly, parenterally administered prostacyclins used to treat severe PAH requires frequent access to clinics and large amounts of provider and financial resources that are not widely available around the world. Efforts should be made to make widely available these diagnostic and therapeutic options available globally. It is also essential to thoroughly consider potential ways by which cultural and economic factors impact the practical diagnostic criteria, therapeutic delivery options, and outcomes of new interventions. It is also essential to closely collaborate with providers who directly work with SchPAH patients.

## Conclusions

A summary of key take-home messages regarding SchPAH is provided in **Table 2**. Despite PAH being an uncommon complication of schistosomiasis infection, the overall high prevalence of schistosomiasis makes SchPAH one of the dominant causes of WHO Group 1 PAH and its severe disease manifestations make SchPAH an important public health threat. The lung blood vessel remodeling in SchPAH follows HSS, which results in embolization of eggs *via* portocaval shunts into the lungs that drives localized inflammation and vascular remodeling. Through a series of cellular and signaling events, Th2 inflammation drives TGF-β activation, which may become autonomous and independent of the *Schistosoma* antigen resulting in persistent vascular disease despite parasite eradication. Taking this into account, it seems logical that targeting the inflammatory cascade might be more beneficial earlier in the disease course (taking into account the need to avoid immunosuppression in individuals who are at risk for recurrent infection) versus targeting TGF-β that may be more beneficial later in the disease course. Treatments that are conventionally used for other forms of PAH appear to be useful in patients with SchPAH, however, without targeting the underling causative mechanisms of the disease. Integrating ongoing clinical studies and translational/basic science approaches to perform mechanistic analysis in animal models, which have clinical relevance to the disease will enable tackling these challenges.

**Table 2:** Key take-home messages regarding schistosomiasis-associated pulmonary arterial hypertension.

- Pulmonary arterial hypertension is thought to occur in about 0.5% of those chronically infected with schistosomiasis, usually following the development of hepatosplenic schistosomiasis. This is an uncommon complication of schistosomiasis, but a major cause of pulmonary arterial hypertension worldwide. - Presenting signs and symptoms of pulmonary arterial hypertension are of heart failure, including shortness of breath on exertion, peripheral edema, and syncope. - Formal diagnosis of pulmonary arterial hypertension requires invasive right heart catheterization. Screening for the disease can be done using echocardiography. - Generally accepted criteria for diagnosis are (must have all 3): o Pulmonary arterial hypertension, currently defined by hemodynamic criteria of a mean pulmonary artery pressure greater than 20 mm Hg, a pulmonary arterial wedge pressure less than or equal to 15 mm Hg, and a pulmonary vascular resistance greater or equal to 3 Wood Units (6th World Symposium on Pulmonary Hypertension, Nice 2018). o History of schistosomiasis infection, as evidenced by current or prior *Schistosoma* parasite eggs in the stool or rectal biopsy, history of prior treatment for schistosomiasis, or exposure to a region where schistosomiasis is endemic o Ultrasonographic evidence of liver disease consistent with HSS, including peri-portal fibrosis and enlargement of the left lobe of the liver. - Anti-helmintics likely slow the rate of progression, but do not reverse established pulmonary vascular disease. Pulmonary vasodilator treatments are available which improve symptoms, but these do not address the underlying disease drivers, and the condition is fatal. - Pathogenic mechanisms by which the clinical disease may develop include: o opening of portocaval shunts around the liver, resulting in: ▪ increased blood flow through the lungs, causing shear stress ▪ egg embolization into the lungs, resulting in mechanical obstruction of the lung vasculature ▪ egg embolization into the lungs, resulting in localized Type 2 inflammation which drives vascular remodeling o a systemic immune response, with Type 2 immunity. - Mice exposed to *Schistosoma mansoni* develop an experimental form of the disease. Experimental pulmonary hypertension does not require the liver disease precursor condition or increased shear stress, and does not occur by mechanical obstruction alone. - A combination of pre-clinical work using animal models and clinical studies has suggested the following series of pathologic events underlies disease pathogenesis: o Type 2 immunity triggered by *Schistosoma* egg antigens, resulting from activation of Th2 CD4 T cells. o Expression of IL-4 and IL-13 results in the recruitment of Ly6c^+^ (classical) monocytes into the lung tissue. o These monocytes express the protein thrombospondin-1 (TSP-1) which can activate TGF-β. o Active TGF-β causes pulmonary vascular disease through promoting pathology in vascular cells. o Pathologic TGF-β signaling may be a final common pathway by which many pulmonary arterial hypertension etiologies cause vascular remodeling; earlier steps may be unique to each specific etiology. o In schistosomiasis, this series of events may function as a negative feedback pathway to decrease excessive host inflammation, and remodeling of the pulmonary vasculature is an unintended, off-target effect. - There are many open questions regarding this disease, which are areas of active investigation.

## Author Contributions

JS, RC, JH, BK, RK, ML, CL, CM, RO, ES, HA, and BG all contributed to writing the initial draft of the manuscript. AC, RC-F, RC, HD, CF, VG, JH, BK, RK, SM, RO, JO-A, LS, ES, and BG revised the manuscript for critical content. All authors contributed to the article and approved the submitted version.

## Funding

RK is supported by the American Heart Association Grant (19CDA34730030) and ATS Foundation/Pulmonary Hypertension Association Research Fellowship; BBG is supported by NIH Grants R01HL135872 and P01HL014985; and CM and BBG are supported by NIH Grant P01HL152961.

## Conflict of Interest

The authors declare that the research was conducted in the absence of any commercial or financial relationships that could be construed as a potential conflict of interest.
